# 3D analysis of Osteosyntheses material using semi-automated CT segmentation: a case series of a 4 corner fusion plate

**DOI:** 10.1186/s12891-018-1975-0

**Published:** 2018-02-13

**Authors:** Rebecca Woehl, Johannes Maier, Sebastian Gehmert, Christoph Palm, Birgit Riebschlaeger, Michael Nerlich, Michaela Huber

**Affiliations:** 1 Department of Trauma Surgery, University Medical Center Regensburg, Franz-Josef-Strauß-Allee 11, 93053 Regensburg, Germany; 2 Faculty of Informatics and Mathematics, Ostbayerische Technische Hochschule, Regensburg, Germany; 3 Department of Orthopedic Surgery, University Clinic Basel, Basel, Switzerland; 4 Department of Neurology, University Medical Center Regensburg, Regensburg, Germany

**Keywords:** 4CF, SLAC wrist, SNAC wrist, Semi-automated segmentation, 3D analysis

## Abstract

**Backround:**

Scaphoidectomy and midcarpal fusion can be performed using traditional fixation methods like K-wires, staples, screws or different dorsal (non)locking arthrodesis systems. The aim of this study is to test the Aptus four corner locking plate and to compare the clinical findings to the data revealed by CT scans and semi-automated segmentation.

**Methods:**

This is a retrospective review of eleven patients suffering from scapholunate advanced collapse (SLAC) or scaphoid non-union advanced collapse (SNAC) wrist, who received a four corner fusion between August 2011 and July 2014. The clinical evaluation consisted of measuring the range of motion (ROM), strength and pain on a visual analogue scale (VAS). Additionally, the Disabilities of the Arm, Shoulder and Hand (QuickDASH) and the Mayo Wrist Score were assessed. A computerized tomography (CT) of the wrist was obtained six weeks postoperatively. After semi-automated segmentation of the CT scans, the models were post processed and surveyed.

**Results:**

During the six-month follow-up mean range of motion (ROM) of the operated wrist was 60°, consisting of 30° extension and 30° flexion. While pain levels decreased significantly, 54% of grip strength and 89% of pinch strength were preserved compared to the contralateral healthy wrist. Union could be detected in all CT scans of the wrist. While X-ray pictures obtained postoperatively revealed no pathology, two user related technical complications were found through the 3D analysis, which correlated to the clinical outcome.

**Conclusion:**

Due to semi-automated segmentation and 3D analysis it has been proved that the plate design can keep up to the manufacturers’ promises. Over all, this case series confirmed that the plate can compete with the coexisting techniques concerning clinical outcome, union and complication rate.

## Background

Scaphoidectomy with midcarpal arthrodesis of the lunate, triquetrum, hamate and capitate was introduced in 1981 by Watson et al. [1] as a salvage procedure for patients suffering from carpal collapse and subsequent arthrosis. The aim of this surgery is to partial preserve motion in the wrist when patients present a symptomatic scapholunate advanced collapse (SLAC) or scaphoid non-union advanced collapse (SNAC) [2]. Various modification of the original operation technique have been described during the last decades using fixation methods like Kirschner wires, staples or compression screws [3–7].

Approximately 50% of the active range of motion and at least 50% of grip strength compared to the contralateral healthy wrist should be preserved after midcarpal arthrodesis to substantiate an adequate outcome independent of the preferred method [8–11].

In 1999 non-locking circular plates specially designed for midcarpal arthrodesis became available. Numerous studies were executed using the non-locking Spider Limited Wrist Arthrodesis System (Kinetikos Medical Inc., San Diego CA, USA). But the initial expectation of generating lower non-union rates compared to the traditional fixation methods could not be verified [4, 12, 13]. Even the development of dorsal locking plates like the carbon based PEEK- Optima plate (Xpode Cup, Trimed Inc., Santa Clarita, CA, USA) or the Variable Angle Locking Intercarpal Fusion System (DePuy Synthes Inc., West Chester, PA, USA) could not provide a conclusive answer which technique results in the least complications [14, 15]. The low profile (1.4 mm) titanium-alloy (TIAI6V4) Aptus 2.0/2.3 for corner fusion plate is another option for the arthrodesis. Despite the low profile advantages of this plate could be the different quadratic shape with a smaller diameter (1.25 cm- 1.45 cm) and more screw options (12) compared to the plate fom DePuy (Diameter 1.5–1.7 cm and 6–7 holes).

A clear advantage of a four corner fusion plate include early mobilization and usually no necessity for follow-up surgery as removing the ostesynthesis material after bony union.

Previous studies evaluated the clinical outcome by measuring pain, range of motion, strength and the radiological outcome by conventional X-ray imaging. It is noteworthy that only few studies considered the multi-slice computerized tomography a valuable asset to screen patients after 4 Corner Fusion [13, 15]. Moreover, limited data exists regarding the configuration of the arthrodesis material itself especially in vivo [16].

In time of digitization new testing methods are required. The present study is designed to determine, whether the Aptus 4 corner fusion plate can match up to the existing methods by assessing not only the clinical outcome but also 3D models generated by semi-automated segmentation of CT scans. The implementation of these new methods allows us to determine coherences between clinical outcome and hardware-associated problems.

## Methods

This study was approved by the institutional review board. Written consent was given by each patient. All patients who received a scaphoid excision and midcarpal arthrodesis using the new 2.0/2.3 Four Corner Fusion (4CF plate) Aptus Plate (Medartis, Basel, Switzerland) from 2011 till 2014 were included in this retrospective case series. Both available sizes, standard and one time the small locking plates were used. The consecutive series of surgeries was performed at our institution by one hand surgeon. Two female and nine male patients gave their informed consent. The surgery was performed in patients with a SLAC wrist (*n* = 8) and a SNAC wrist (*n* = 3), whereas the dominant hand (n = 8) as well the non-dominant hand (n = 3) were affected. In one female we used the small size after availability due to the anatomically small wrist. We had the impression in some cases, that the plate with the diameter of 14.5 mm was to big for some wrists.

### Surgical technique

A dorsal classical Berger approach was used to open the wrist [17] and the scaphoid was excised. The cartilaginous surface was removed from the midcarpal joint and the surface between the capitate and hamate before the lunate, capitate, hamate and triquetrum were temporarily fixated with K-wires. Afterwards, the plate bed was reamed free-handed as deep as required to avoid impingement of the plate. Finally, cancellous bone graft from the scaphoid was used to minimize the gaps between the carpal bones. Afterwards the four corner fusion plate was inserted and fixed with screws. A final survey with fluoroscopy was performed, checking for plate position, screw length and impingement during passive movements. A dorsal splint was applied and active assisted physiotherapy started on the first day postoperatively. A removable orthesis was applied after approximately two weeks when soft tissue swelling returned to normal conditions and supported immobilization for the following four weeks.

The active range of motion (ROM) was measured preoperatively, three weeks, six weeks and six months after surgery. Grip and pinch strength were assessed with a dynamometer at the follow-up dates. Additionally, the quality of life was determined by describing pain at rest and in motion on a visual analogue scale (VAS, 0 equalling no pain, 10 equalling the maximum of pain). The Disability of the Arm, Shoulder and Hand questionnaire (DASH) [18] and the Mayo Wrist Score [19] had to be completed by all patients.

Lateral and posteroanterior radiographs were taken preoperatively and postoperatively to access the placement of the plate, determine the severity of carpal collapse by measuring the scapholunate angle and to document any complications.

Six weeks after surgery a CT scan of the wrist was performed to allow a first statement about union and to record hardware position. A new protocol was developed which relies on semi-automated segmentation and the 3D analysis of the emerged models to gain additional information about osteosynthesis material postoperatively [20]. The dicom data of the CT scans were implemented with a segmentation tool (ITK Snap, Open Source, Version 3.4.0) [21]. To generate three-dimensional surface meshes of the four carpal bones, the plate and the screws the axial CT layers were used to define a region of interest (ROI) by setting upper (2000 Hounsfield Units, HU) and lower (220 HU) threshold limits for the carpals or rather a lower threshold for the osteosynthesis material (2000 HU). In addressing each structure by placing circles of different size into the ROI, the active contour segmentation automatically transferred these circles into 3D bubbles. These bubbles must be set anatomically correct, because if they exceed the contour of the bone in only one layer the small cartilaginous border is overstepped and consequently two different structures are treated as one. After checking and location adaption of the 3D bubbles the segmentation evolved automatically. If the iteration count is chosen too big, the structures also merge into each other as described above. If it is chosen too small, holes occur, so that the ideal iteration count lies between these two extreme values. After semi-automated segmentation and manual corrections the resulting surface meshes were imported into another software (Meshlab, Open Source, Version 1.3.3) [22] designed for direct mesh analysis. By visualization of the 3D environment, the union of the arthrodesis and the multidirectional position of the screws were surveyed. To demonstrate the bicortical ply of the screws a uniform mesh resampling filter was used to create the inner shell of the corticalis from the outer shell by remeshing the surface mesh of the four carpals. After repairing the three-dimensional meshes with Meshlab by using filters for cleaning and creating a watertight mesh, physical dimensions and capacities as well as the relations between anatomical structures and osteosyntheses material were determined by the software netfabb Basic (netfabb Basic, netfabb GmbH, Lupburg, Germany). Measuring the screw length accurately the software 3D Tool Free Viewer (3D–Tool GmbH & Co. KG, Weinheim, Germany) was further utilized. For visualization the surface meshes were post processed with depth and laplacian smoothing filters and coloured using the RGB colour code for bone and metal. Data exchange between different mesh-related software tools was organized by the stereolithograhy (stl) format. All these clinical, radiographical and three-dimensional results were compared and screened for complications.

## Results

### Clinical outcome

Between August 2011 and July 2014 eleven patients (9 men and 2 women) received a four corner fusion with the 4CF plate for eleven wrists (7 right, 4 left). Their mean age was 49 years [range, 32–69 y]. The trauma that led to the SLAC (8) or SNAC (3) wrist happened on average thirteen years [range, 1–40 years] ago. All patients completed the required six month follow up. After an average of 113 days [range, 59–191 days] patients returned to their jobs.

The mean active range of wrist motion prior to operation was determined with 40° extension and 42° flexion. After midcarpal arthrodesis the wrist motion was limited to 30° extension and 30° flexion which correlated with an average of 54% extension and 48% flexion compared to the unaffected hand. In addition, 78% radial and 55% ulnar deviation remained after surgery when compared to the unaffected side whereas pronation and supination were preserved at all.

The mean static grip strength was significantly (paired t-test: *p* = 0,014) increased from 10 kg [range, 1–20 kg] to 18 kg [range, 18–55 kg]. The grip strength reached 54% and the pinch strength reached 89% of the force of the unaffected wrist (see Table 1).

**Table 1:** Clinical Outcomes

Variable	Preoperative	Postoperative
Extension [°]^a^	40 ± 23	30 ± 15
Flexion [°]^a^	42 ± 19	30 ± 9
Radialabduction [°]^a^	19 ± 9	18 ± 7
Ulnarabduction [°]^a^	18 ± 6	16 ± 6
Grip strength [kg]^a^	10 ± 7	18 ± 10
Grip strength [%]^a, b^	30 [9–51]	54 [24–84]
Pinch strength [kg] ^a^	7 ± 3	8 ± 4
Pinch strenght [%]^a, b^	78 [29–93]	88 [84–89]

^a^expressed as mean ± SD

^b^compared to the contralateral healthy wrist

Mean preoperative pain values under resting conditions revealed 4 and under stress conditions 7 on a Visual Analogue Scale (VAS). 0 on the VAS equals no pain whether 10 equals the maximum of pain. A significant decrease of postoperative pain values to 1 at rest and 4 at stress was noticed by patients (paired t-test: *p* = 0,024; p = 0,018). In addition, four patients reported a complete pain relief and five patients experienced solely slight pain under job-related work.

The mean contempt (surveyed in the Mayo Wrist Score) significantly ascended (paired t-test: p = 0,006) from 11 (± 9) points) prior to operation to 21 (± 4) after surgery. The average DASH score at the six month follow up was 31 (± 21) which was significantly reduced when compared to the preoperative value of 50 (± 14) (paired t-test: p = 0,007).

### CT analysis

**Fig. 1:**
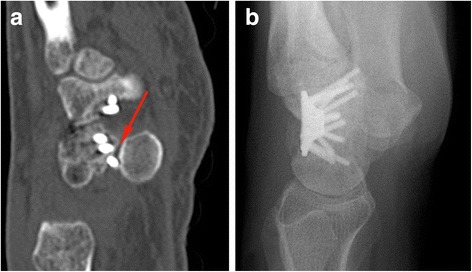
Only the CT scan (**a**) revealed a screw displacement into the pisotriquetral joint (see arrow), which was not obvious on conventional X-Ray [**b**, sagittal view]

Ten patients received the standard size plate and one patient was served with a small size plate for the four corner fusion. Union was detected in the postoperative CT scan in all patients six weeks after midcarpal arthrodesis. No evidence of broken or loosened hardware was apparent in the CT scan and no signs of a progressing arthrosis was verified. Nonetheless, pathologies were determined in two cases as follows:

A screw affected the pisotriquetral joint in one patient which was revealed by the CT scan (Fig. 1 a). The patient complained about rest pain, which increased during motion and strain. Extension and flexion of the wrist showed an impaired active motion during all follow up meetings. The postoperative conventional X-ray images (Fig. 1b) did not provide any evidence of screw displacement.

**Fig. 2:**
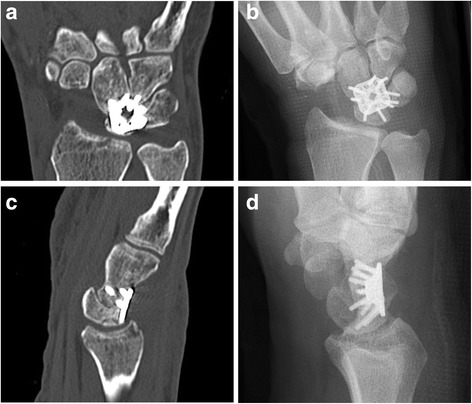
The plate position appears to be correct in the initial review of the X- Ray **b**: anteroposterior view, **d**: sagittal view], but the CT scans (**a, c**) revealed a proximal pate placing

In another case, the CT Scan (Fig. 2 a, c) showed a proximal positioning of the plate which was associated with pain after stressful wrist motion. In addition, the wrist extension was restricted to 20° six weeks postoperative. The proximal position of the plate can also be seen in the conventional X-rays when provided with the information of the CT scan (Fig. 2 b, d).

**Fig. 3:**
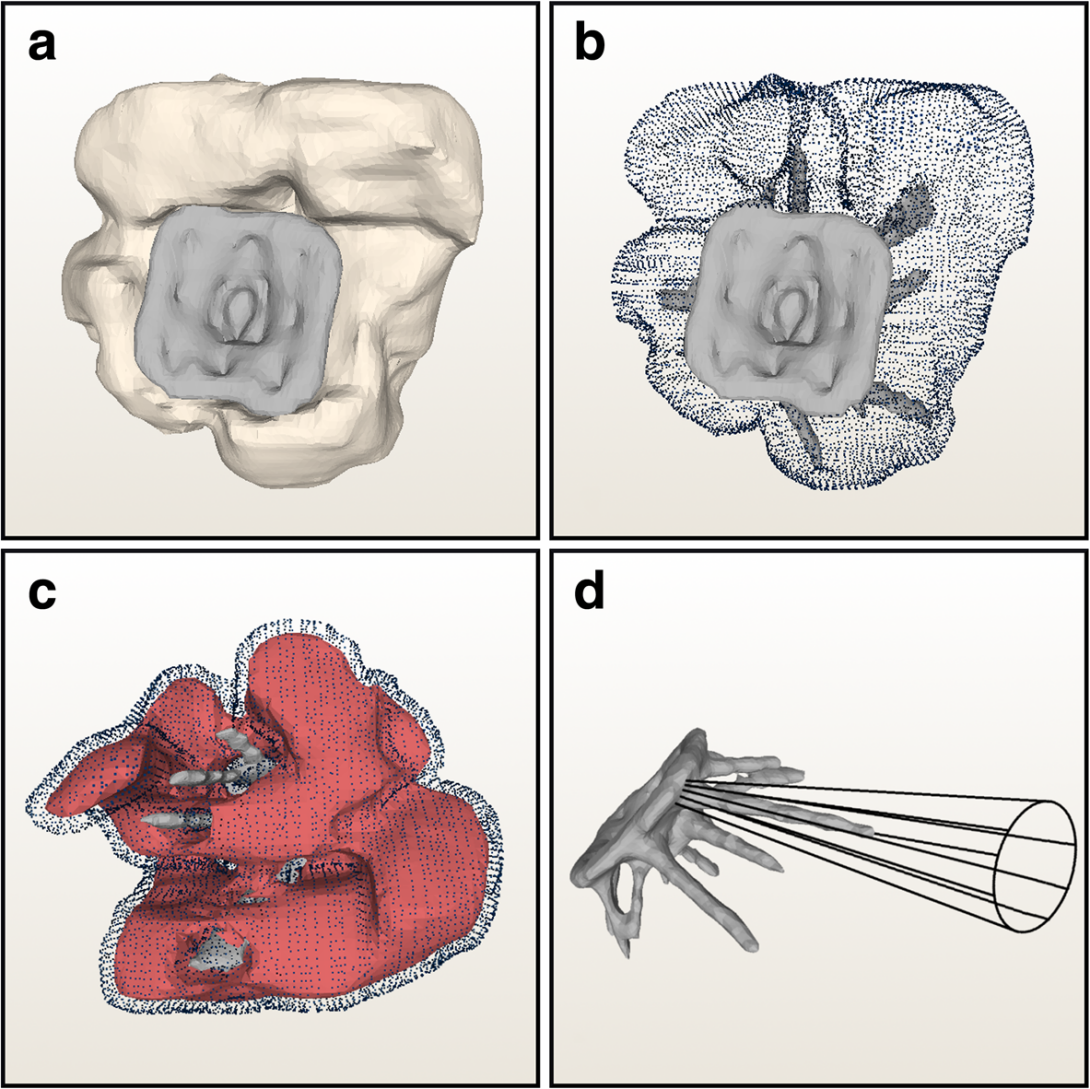
The models of the four carpals fused (white) and the plate (grey) were tested in a three-dimensional environment (**a**). Distinguishing the screw count in each carpal from a sole anteroposterior view (**b**) was not feasible, but a 360° view and a dynamic examination allowed an exact attribution. To visualize the bi-cortical ply (**c**) a mesh was produced symbolizing the inner layer of the cortical (red). Only screws, which penetrated the red shell were distinct bi-cortical located (**d**). The multidirectional angle of 15° was established, if the screw lay in a cone (black) with the same defaults and in the direction of the normal vector of the plate

*3D Analysis -* Reviewing the 3D meshes the progressing union of the four carpals was proven as well (Fig. 3a). In all but one of cases at least two screws could be used as a fixation for the lunate, triquetrum, hamate and capitate (Fig. 3 b). The sole exception occurred concerning the small size plate. Due to the anatomic proportions, only one screw could be placed into the hamate. Working with the standard size plate the placement of even three screws was able in eight of ten cases respectively concerning one carpal. Observing the cortical ply, it could be established that on average 7 (± 3) screws lay bicortical (Fig. 3 c). The multidirectional angle of 15°, which is necessary to ensure locking, could be substantiated for all 125 screws employed (Fig. 3 d).

The dimensions of the four carpal bones after arthrodesis revealed a length of 3.67 cm [range 3.30–4.40] and a width of 3.41 cm [range 2.91–3.84] whereas the standard plate provides a length and width of 1.45 cm and 1.25 cm for the small size plate. Therefore, the ratio between plate and carpal bones represents 42% of the length and 40% of the width which allows the surgeon sufficient options for placement.

The distance between plate and proximal carpal bones was measured to specify the required distance between radius and arthrodesis material to avoid impingement. The mean distance between the margin of the plate and the lunate was 6.1 mm [range 2.4–10.6] and to the triquetrum 8.2 mm [range 1.5–11.9].

The mean volume of the plate and screws reached 0.66 cm^3^ [range 0.47–0.71] and the volume of the four fused carpals obtained a mean volume of 10.62 cm^3^ [range 6.29–16.05]. The approximate loss of bony substance due to the fusion showed an average of 6.2% [range 4.4–7.5].

## Discussion

Hence scaphoidectomy and midcarpal arthrodesis are designed as salvage procedures their main goal is pain reduction while preserving wrist movement and grip strength. Palmer et al. defined the functional wrist motion with 30° extension, 5° flexion as well as 10° radial abduction and 15° ulnar abduction [23].

A mean active arc between 42% and 59% compared to the contralateral wrist is reported in previous studies when using 4 CF with traditional methods [6–8, 24]. These results are similar to midcarpal arthrodesis accomplished by compression screws or staples which a documented range from 50% to 54% [3–5, 10]. However, studies from Ashmead and Dacho showed a non-union quote of a minimum of 3% [8] and maximum 10% [6]. Focussing on hardware associated complications for dorsal impingement the rate ranged from 3% [4] and 13% [8]. The complication rate increased up to 23% [8] when general complications (e.g. infection, CRPS) were considered as well.

Various studies reported preserved motion between 45% and 70% compared to the unaffected healthy wrist when using a non-locking Spider plate [12, 13, 25]. The union proportions for the Spider plate was 100% in the study of Merrell et al. compared to a non-union rate of 62,5% in the study of Kendall et al. [8, 9, 24]. An explanation of this result could be the fact that only eight patients were included in the study of Kendall, whereas five patients experienced a non-union However, complications concerning hardware failure, dorsal impingement and pisotriquetral joint penetration was reported for 7% [12] and 29% [4] of the patients.

The PEEK- Optima plate could attend with similar results. On average 63% of extension and 51% of flexion were measured [15, 26, 27]. Fusion was achieved in 80% to 96% of all cases indicating that the locking mechanics resulted in higher union rates. Most complications were related to hardware or impingement.

Extension and flexion remained at 30° compared to the healthy wrist when using the Four Corner Fusion Aptus Plate. A significant improvement in grip strength was noticed, while pain at rest, in motion and under stress showed a significant decrease. All patients reported a positive impact on their quality of life after surgery (cf. DASH score). The overall complication quote after midcarpal arthodesis with the Aptus plate reached 18% due to dorsal impingement (*n* = 1, 9%) and hardware associated complications (n = 1, 9%). This study showed an excellent fusion rate of 100% in the CT scans six weeks after surgery despite no other cancellous bone graft than the scaphoid was used. Tielemans et al. could even prove that no bone graft at all is necessary to ensure fusion one year after four corner fusion [28]. Merrell et al. proposed that one reason for non-union might be the use of the sclerotic scaphoid bone [13]. However, the distinct biomechanics of the locking plate might be primarily the reason for the bony union whereas bone grafts only play a minor role based on the present and previous studies [16].

The key to defining the success of a midcarpal arthrodesis is to proof the presence of a bony union after surgery which has to be proven [27], no reliable characteristics exist to define radiographic union [6].

Bedford and Yang defined the absence of clear signs of non-union e.g. loosening of hardware as fusion of the bones [12]. Rhee and Shin relayed on a combination between lack of pain and the radiographic union of the capitate and lunate as a marker for union. In cases of uncertainty the authors initiated a supplemental CT scan. There are only a few clinical trials which included CT scans into the original protocol even though it is proven that union can be detected early and sufficient [29–31].

To our knowledge, to date no other study provide evidence of bony union after arthrodesis using a semi-automated segmentation and 3D models. The described method was utilized to verify bony union. We could provide in vivo data that the small size plate and the regular size plate allow the application of at least two screws per carpal bone if the correct position of the plate was ensured. In addition, we could quantify the loss of bony substance due to the reaming for plate placement and provided evidence that this reduction did not affect the consolidation time of six weeks postoperatively even though unloaded mobilisation was started immediately after surgery. All locking screws reached the multidirectional angle of 15°. Based on the different angles screw length could markedly vary even for one carpal bone, which made an intraoperative fluoroscopy essential to avoid complications by joint penetration. Another advantage of locking screws became evident when even a mono-cortical fixed screw did not interfere with the bony fusion of the four carpal bones. The screw length always should be chosen rather short than long bearing in mind that overlong screws can cause pain and thereby restrict wrist motion. Moreover, the correct placement of the plate is mandatory to ensure an acceptable pain reduction for the patient after surgery. According to the producers’ guide the ream should be positioned in the centre of the hamate, lunate, triquetrum and capitate freehand. However, the authors found it quite difficult since the instrument can distort during reaming. The three-dimensional reconstructions showed that even if the plate was countersunk below cortical level a non-centred placement of the plate could lead to dorsal impingement. Taken together, it would be preferable when the manufacturer provide a reaming guide similar to the one which is already in use for the Variable Angle Locking Intercarpal Fusion System [14]. Thereby, this crucial operation step could be simplified while ensuring proper placement of the arthrodesis material. Beside the position of the plate the selection of the fitting size is very important. If a preoperative CT scan of the wrist is available, the surgeon can measure the size of the carpals in the multiplanar reconstruction or by using 3D analysis. Otherwise the surgeon should choose intraoperatively pondering between stability of the four corner fusion and the risk to create an impingement.

### Limitation

The limitations of this study are the small number of patients and the short follow up period of 6 moths. However, this study was designed as a pilot project to assess whether CT scans, semi-automated segmentation and the creation of 3D models added additional value to test new osteosynthesis methods.

## Conclusion

Overall the new plate can compete with other fixation methods for midcarpal arthrodesis concerning postoperative motion, strength, pain reduction, union and complication rate. The results of this study suggest, that CT scans should be conducted on a more regular basis, especially if patients report about postoperative pain or restrictions in movement. With the aid of semi-automated segmentation and 3D analysis we were able to evaluate the hardware and its associated problems in vivo. In principle this method can be used to analyse all kind of bone-metall interaction, if a specific test protocol is established for the system to be tested.

To date, segmentation is still a time-consuming practise which restrict the clinical application to studies and selected issues e.g. testing new osteosynthesis techniques, to gain further informations beyond the established methods. However, when fully-automated segmentation appears on the software market it might be possible that three-dimensional models can be established as a clinical routine procedure to serve surgeons before and after surgery.

## Abbreviations

3D: Three dimensional
4 CF: Four corner fusion
CT: Computerized tomography
DASH: Disabilities of the arm, shoulder and hand
et al.: Et alii/et aliae
ROM: Range of motion
SLAC: Scapholunate advanced collapse
SNAC: Scaphoid nonunion advanced collapse
stl: Stereolithography
VAS: Visual analog scale
